# Yellow-Gold Polarized Light Microscopy May Improve Accuracy of Pathological Staging of Colorectal Adenocarcinoma

**DOI:** 10.7759/cureus.9007

**Published:** 2020-07-05

**Authors:** Yana Puckett, Ruc Tran, Mitchell Wachtel

**Affiliations:** 1 Surgical Oncology, University of Wisconsin, Madison, USA; 2 Surgery, Texas Tech University Health Sciences Center, Lubbock, USA; 3 Pathology, Texas Tech University Health Sciences Center, Lubbock, USA

**Keywords:** microscopy, colon cancer, rectal cancer, adenocarcinoma, polarized light, pathology, staging, yellow light

## Abstract

Introduction

Polarized light (PL) has been used in pathology for multiple reasons, including the demonstration of foreign bodies, the evaluation of crystals, and the demonstration of fibrosis. We incidentally found that yellow-gold polarization routinely occurs surrounding desmoplastic scar tissue abutting the invasive glands of colonic adenocarcinoma. We hypothesized that evaluating the use of polarized light over a series of invasive adenocarcinomas of the large intestine might produce evidence of its utility.

Methods

Large intestinal resections with invasive adenocarcinoma were reviewed with yellow-gold polarized light microscopy by two surgical pathologists postoperatively between January 2017 and March 2019. Specimens were examined under yellow-gold polarized light to evaluate invasion from the submucosa into the muscularis propria, from the muscularis propria into pericolic fat, and to the serosa. The diagnosed location, T stage, history of radiotherapy, mucinous features, and grade were recorded. Photographs were taken when images were deemed to be of value. The two-tailed Fisher’s exact test was used to compare the invasion detection of the tumor into fat in scar tissue in colorectal cancer.

Results

A total of 75 large intestinal resections with invasive adenocarcinoma from 75 patients were accessioned. Concerning the initial stage, three (4%) were T1, nine (12%) were T2, 46 (61%) were T3, and 17 (22%) were T4. A history of previous radiation treatment was seen in 10 (13%). Two (2%) were poorly differentiated. Nine (12%) were mucinous carcinomas; mucinous areas were seen to pose difficulty in 12 (16%). Overall, one out of nine, initially staged as T2, was upstaged to T3 (11%), with the addition of yellow-gold polarized light microscopy. One tumor was downstaged from T2 to T1 (11%). For many T2 and T3 tumors, invasion into the muscularis propria was better defined by yellow-gold polarized light.

Conclusion

Yellow-gold polarized light microscopy may be a useful adjunct to conventional microscopy in more precisely staged pathological colorectal cancer specimens.

## Introduction

Pathologists frequently encounter difficulties in interpreting the invasion of adenocarcinoma on slides under microscopy for colorectal cancer after the removal of the specimen by the surgeon. The most difficult issues in pathologic staging involve an assessment of transmural tumor extension (pathologic (p) tumor (T) in situ (is) versus (vs) pT1, pT1 vs pT2, pT2 vs pT3, and pT3 vs pT4) and an analysis of regional lymph node status [1-2].

Multiple studies have shown polarized light microscopy is a technique that helps better delineate structural alterations in biological tissue, including cancer [3-7]. We incidentally found that yellow-gold polarization microscopy routinely occurs in desmoplastic scars abutting the invasive glands of colonic adenocarcinoma. We hypothesized that evaluating the use of polarized light over a series of invasive adenocarcinomas of the large intestine might produce evidence of its utility.

Polarized light is a technique that has been present over the last two decades. It is readily available for pathologists and consists of noninvasive two-dimensional (2D) imaging of histological structures. It is also a useful adjunct in microscopy, with multiple diagnostic applications [4,7-9]. Crystals, natural and artificial fibrous structures, pigments, lipids, proteins, bone, and amyloid deposits exhibit birefringence or the ability to produce polarized light. In histology, birefringence is produced by asymmetric particles too small to be resolved even by the best lenses [4,7-9].

The polarizing microscope converts all passing light into vibrating light in one optical plane. If birefringence is not exhibited by the particle, the field will remain black. If, however, there is birefringence, the particle will appear bright upon the dark background. This happens when a birefringent crystal splits a ray of light into two paths at right angles to each other, causing vibration of the light in an optical plane different from that of the incident light. Weak birefringence can be enhanced through the use of dyes such as the addition of Congo red for amyloid or impregnating metals in an orderly linear alignment [4,7-9].

Tumor invasion appears differently due to the difference in the birefringent properties of the epithelium and fiber connective-tissue structures. As the tumor develops, the tissue structure changes. Polarized light can detect neoplastic processes and reorganization of structures with high sensitivity [7]. The difference in birefringent properties is enhanced by yellow-gold polarized light and appears as a bright yellow invasion on microscopy.

The specific aim of this study is to see whether yellow-gold polarized light microscopy can improve the accuracy of pathologic staging in colorectal cancer.

## Materials and methods

Colorectal intestinal resections with invasive colorectal adenocarcinoma proven by pathology on colonoscopy were selected. A total of 10 individual surgeons performed the resections between January 2017 and March 2019 at an academic institution under regular microscopy and yellow-gold polarized light. All patients treated surgically for colorectal cancer during that time frame were selected for the study. Yellow-gold polarized light was used to look for invasion from the submucosa into the muscularis propria, from the muscularis propria into pericolic fat, and to the serosa. Specimens with abscesses or perforations were excluded from the study. Diagnosed locations, T stage, history of radiation treatment prior to surgery, mucinous features, and grade were recorded. Photographs were taken when images were deemed to be of value. Two board-certified surgical pathologists reviewed the resections for congruity of findings. The pathologists were blinded to each other's interpretations.

All slides were hematoxylin and eosin (H&E) stained. Slides with tumors were evaluated to find difficult foci with respect to the invasion of the muscularis propria, pericolic tissue, or serosa (obscured by scar or inflammation), which might be suitable to examination by yellow-gold polarized light. Particular attention was devoted to the invasion of the muscularis propria from the submucosa, to invasion beyond the muscularis propria, to the definition of the muscularis propria where this might prove difficult, and to the involvement of the serosa. Areas of interest were evaluated at the 2.5X objective through a Leica 170HD camera (Leica Camera AG, Wetzler, Germany) attached to a Leica DM 2000LED microscope (Leica Camera AG) with and without yellow-gold polarized light. The camera proved important because it automatically brightened the images darkened by polarizing filters, thus permitting repetitive comparisons between polarized and nonpolarized light.

## Results

A total of 74 colon resections were evaluated, which included a total of 75 tumors (one specimen bore two contiguous cancer lesions). Table 1 displays the frequency distributions of tumor stages and the locations of tumors.

**Table 1:** Frequency distribution of site and T stage of 75 invasive colorectal adenocarcinomas

	N (%)
Tumor site	
Cecum	14 (18.7%)
Cecum-Ascending	1 (1.3%)
Ascending	10 (13.3%)
Transverse	7 (9.3%)
Transverse-Splenic	1 (1.3%)
Transverse-Descending	1 (1.3%)
Descending	3 (4%)
Sigmoid	19 (25.3%)
Rectosigmoid	8 (10.7%)
Rectum	11 (14.7%)
	75 (100%)
T stage	
T1	3 (4%)
T2	8 (10.7%)
T3	47 (62.7%)
T4	17 (22.7%)
	75 (100%)

A history of changes due to radiation treatment prior to surgical resection was seen in 10 (13%). Two (2%) were poorly differentiated adenocarcinomas. Nine (12%) were mucinous carcinomas. Overall, one out of nine initially staged as T2 was upstaged to T3 (11%) with the addition of yellow-gold polarized light microscopy. One tumor was downstaged from T2 to T1. The proportions of the left and right colon tumors were not related to T-stage proportions (P>0.50, Fisher’s exact test two-tailed).

The cancers evaluated in this cohort were evenly distributed in terms of location within the colon. Approximately 14.7% of the specimens evaluated were of the rectum. Of the 75 specimens evaluated, the overwhelming majority of specimens (62.7%) were diagnosed as stage T2 after pathologic evaluation.

## Discussion

**Figure 1:**
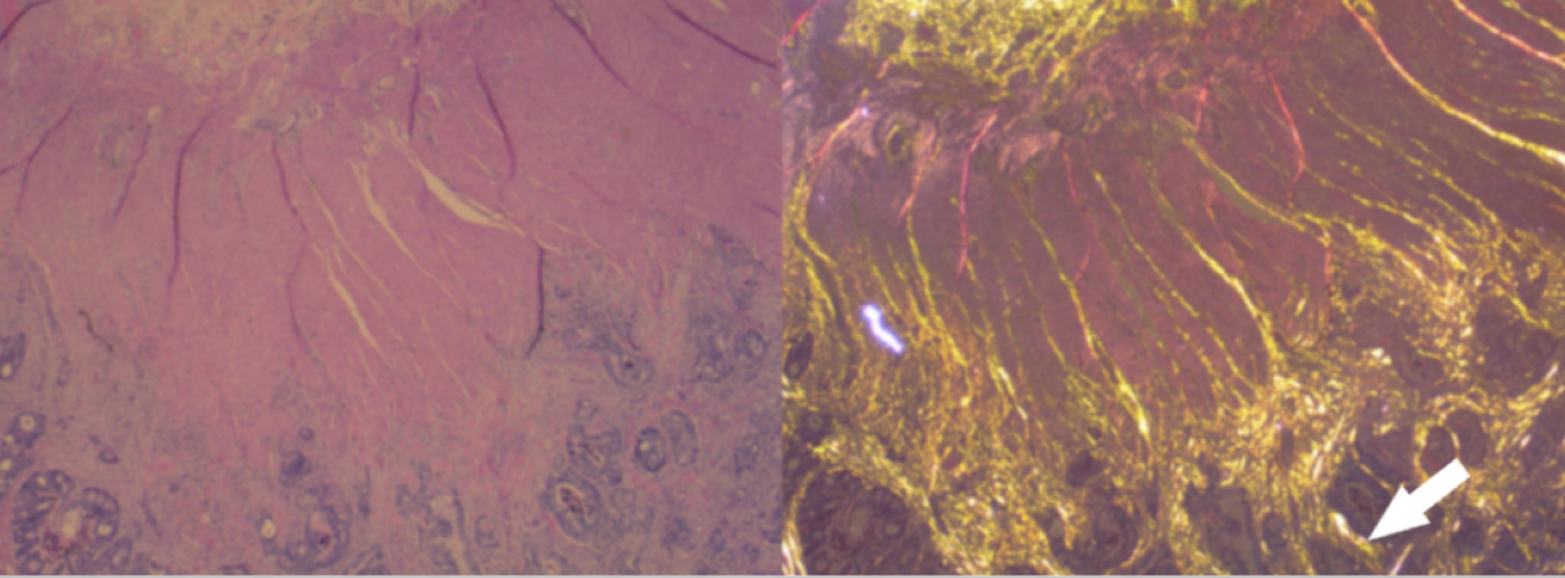
Example of how yellow-gold polarized light microscopy demonstrates and better elucidates invasion into the muscularis propria from the submucosa Invasion can be seen as the yellow-gold light, which can be better seen invading into the muscularis propria from the submucosa (white arrow).

Our series showed that one tumor initially diagnosed as T2, which assuredly appeared to be so on conventional H&E examination, was found to be T3 upon examination with yellow-gold polarized light (Figure 1).

Yellow-gold polarized light was also helpful in ruling out invasive nature in those equivocal foci thought to be invasive that turned out to be non-invasive upon yellow-gold polarization. In addition, in most cases, yellow-gold polarized light microscopy provided clarity and allowed the pathologists a more definite assignment of the T1, T2, and T3 stages. However, it was of no assistance with respect to identifying T4 tumors, as the serosa is poorly visualized with yellow-gold polarized microscopy. For many T2 and T3 tumors, invasion into the muscularis propria was subjectively noted to be better defined by yellow-gold polarized light.

**Figure 2:**
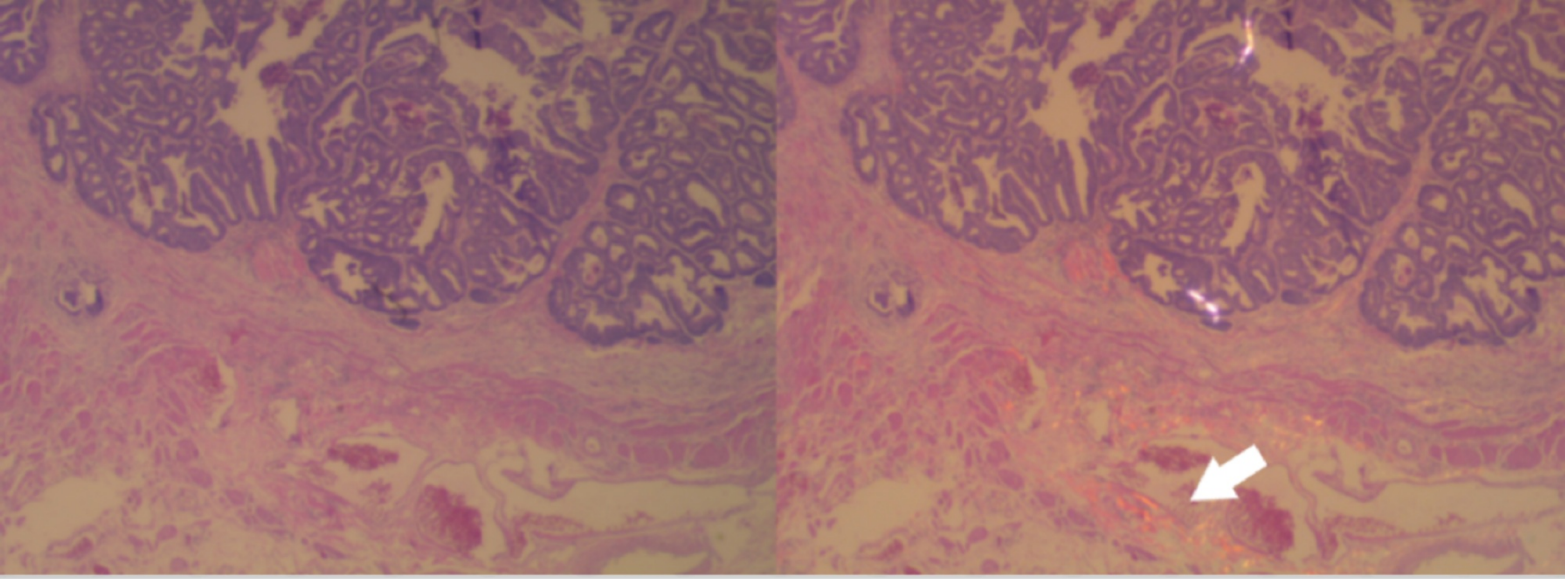
Invasion first thought to be T2 correctly downstaged to T1 with yellow-gold polarized microscopy (white arrow)

**Figure 3:**
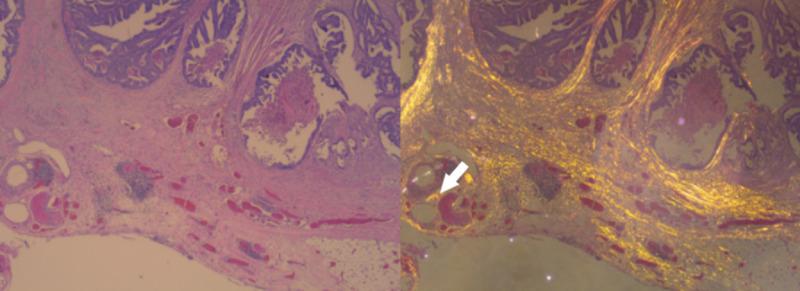
Case first thought to be T2; however, upstaged to T3 with yellow-gold polarized light The yellow-gold color can be seen invading into the pericolic fat, which establishes the T3 stage (white arrow).

Thus, we incidentally discovered that polarized yellow-light microscopy may be sensitive to picking up tumor invasion into fat in colorectal cancer after surgical resection. We found that, overall, one out of nine initially staged as T2 was upstaged to T3 (11%) with the addition of yellow-gold polarized light microscopy (Figure 2). One tumor was downstaged (Figure 3) from T2 to T1.

We observed during the study that invasive glands were observed to incite a desmoplasia, comprising of young fibroblasts with clear or light blue-tinged spaces. The surrounding desmoplasia was observed to be replaced by ordered fibroblasts that polarized gold to yellow. The smooth muscle in muscularis propria was observed not to polarize but occasionally polarized pink or orange. The submucosa, stroma bounding vertically oriented blood vessels within the muscularis propria, and serosa/pericolic adipose tissue all showed material that polarizes gold to yellow, usually in thin streaks. This was the case except in specimens that were distorted by previous radiation treatment, operative changes of biopsy, or adhesions. Juxtaposition under polarized light of yellow or gold material to cancerous glands or desmoplasia thus was visualized to locate at the level of invasion with respect to the muscularis propria.

True invasion of the muscularis propria from the serosa showed a jagged yellow-gold edge (Figure 1). Muscle that either does not polarize or polarizes light pink or orange can present a false impression of invasion of fat. It was observed that the invasion of fat can be identified as gold yellow juxtaposed to desmoplasia. Gold yellow pericolic stroma can be visualized to surround invading glands just beyond the edge of the muscularis propria. The polarizable scar was observed to extend directly from the glands into pericolic fat.

The utility of the technique made itself evident upon examination of a difficult case seen after completion of the study. A tumor lacking, on initial H&E examination, was considered not to show invasion into pericolic fat, which seemed odd, given an associated 1.5 cm lymph node that was positive for invasion. Although most of the lesion failed to show the invasion of pericolic adipose tissue (Figure 2), a focus within the desmoplastic scar extending into fat was seen. Unknown from this analysis is the proportion of tumors initially thought to be an early T3 stage, which would be called the T2 stage upon yellow-gold polarized light examination. Cologuard testing, now used with more frequency, produces earlier stage tumors at resection [10]; the difficulties associated with distinguishing early T3 tumors from T2 tumors are likely to increase over time. As such, polarized light microscopy may be a helpful adjunct.

Other studies depict the use of polarized light microscopy in tumor invasion analysis. One study performed by the Harvard Medical School dermatology department revealed a combination of polarized light microscopy with conventional histological analysis of nonmelanomatous cancer cells. There was a better detection of an in-situ tumor with decreased time and cost [11]. Another study used polarized-light microscopy in the depiction of melanocytic lesions. The study was performed by Seidenari et al. in 2003. Their aim was to develop a computerized method for the identification of color areas in melanocytic lesions through the use of polarized light microscopy in hopes of automating the detection of melanomas more readily [5,11].

Polarized light was also studied in pre-cancer detection in cervical cancer by Sokolov et al. Their study sought a way to detect cancer in precancerous lesions without an invasive biopsy by looking at cervical epithelial cancer cells. Their study showed promising preliminary results, however, no comparison group was established [6].

It is unclear why polarized light microscopy is able to detect invasion better than conventional histologic microscopy, however, one theory could be that fibrosis may have a relationship with tumor invasion. Because polarized light may be better at detecting fibrosis than conventional microscopy, it is plausible that this is the reason polarized light can detect tumor invasion with higher precision when used as an adjunct. A study by Juan Rosai in 1981 alluded to the fact that there may be a relationship between pancreatic fibrosis and the pseudoneoplastic proliferation of endocrine cells [12]. However, further research is needed to verify this relationship.

According to the National Comprehensive Cancer Network (NCCN) guidelines, the adjuvant treatment for colon cancer is based on the pathologic stage of the colon. The adjuvant treatment for node-negative T1 and T2 colon cancer is observation and surveillance. However, the treatment for high-risk, node-negative T3 colon cancer may include adjuvant chemotherapy [13]. The addition of chemotherapy to high-risk T3 colon cancer is associated with longer survival [13]. As such, yellow-gold polarized light microscopy may have implications for increased accuracy of pathological colon cancer staging and thus improve the accuracy of treatment for patients and may have better survival implications. The implications for rectal cancer and yellow-gold polarized light microscopy are still unclear and warrant further investigation.

The strength of our study is that it is easily replicable with minimal time consumption. One can, with a microscope, camera, and polarizing lens in place, assess a low-power field under polarized light in under 10 seconds. There is no cost involved in the use of polarized light microscopy, and it is readily available in most laboratories.

The limitations of our study are fivefold. First, we lacked tumors that were early T3, precluding any definite statements about the use of polarized light to ensure T2 tumors were not underdiagnosed. Second, there were only three T1 tumors, meaning that nothing could be definitely said about using this technique to distinguish such tumors from those that invade muscularis propria. Third, the use of this technique in cases where patients had undergone prior radiation treatment needs to be more carefully assessed as tissues associated with radiation were laden with increased fibrosis and, thus, may render the microscopy inaccurate. Future studies looking into yellow-gold polarized light would benefit from excluding patients with previous radiation treatment. This study is observational in nature. Further randomized controlled studies should be performed to confirm the enhanced accuracy of the staging of yellow-gold polarized light microscopy. Finally, we did not evaluate the utility of this technique in distinguishing invasive carcinoma from high-grade dysplasia on biopsy, assuredly a focus of future interest and research.

## Conclusions

Yellow-gold polarized light microscopy is a readily available and affordable adjunct to regular microscopy. It is available in most laboratories. Our investigators incidentally found that yellow-gold microscopy enhances the clarity of adenocarcinoma invasion in colorectal cancer. It was found to be helpful in establishing diagnoses with more assurance. In addition, it was seen to upstage one tumor in the cohort from T2 to T3 and downstage one tumor from T2 to T1. Randomized control trials are needed to demonstrate this effect more definitively. However, in the meantime, it may enhance the accuracy of the pathological staging of colorectal cancer and may be a useful adjunct to conventional microscopy in more precisely staged pathological colorectal cancer specimens.

## References

[1] Liu Q, Luo D, Cai S, Li Q, Li X (2018). P-TNM staging system for colon cancer: combination of P-stage and AJCC TNM staging system for improving prognostic prediction and clinical management. Cancer Manag Res.

[2] Compton CC (2006). Key issues in reporting common cancer specimens: problems in pathologic staging of colon cancer. Arch Pathol Lab Med.

[3] Aronson JF (1984). Demonstration of lung type II cell differentiation by polarized light microscopy. Dev Biol.

[4] Oldenbourg R (2013). Polarized light microscopy: principles and practice. Cold Spring Harb Protoc.

[5] Pellacani G, Grana C, Seidenari S (2004). Automated description of colours in polarized-light surface microscopy images of melanocytic lesions. Melanoma Res.

[6] Sokolov K, Nieman LT, Myakov A, Gillenwater A (2004). Polarized reflectance spectroscopy for pre-cancer detection. Technol Cancer Res Treat.

[7] Wolman M (1975). Polarized light microscopy as a tool of diagnostic pathology. J Histochem Cytochem.

[8] Cole WV (1948). Polarized light in photo-microscopy. J Biol Photogr Assoc.

[9] Swann MM, Mitchison JM (1950). Refinements in polarized light microscopy. J Exp Biol.

[10] Yaroslavsky AN, Barbosa J, Neel V, DiMarzio C, Anderson RR (2005). Combining multispectral polarized light imaging and confocal microscopy for localization of nonmelanoma skin cancer. J Biomed Opt.

[11] Bartow SA, Mukai K, Rosai J (1981). Pseudoneoplastic proliferation of endocrine cells in pancreatic fibrosis. Cancer.

[12] Berger BM, Schroy PC, Dihn TA (2016). Screening for colorectal cancer using a multitarget stool DNA test: modeling the effect of the intertest interval on clinical effectiveness. Clin Colorectal Cancer.

[13] Iveson T, Sobrero AF, Yoshino T (2019). Prospective pooled analysis of four randomized trials investigating duration of adjuvant (adj) oxaliplatin-based therapy (3 vs 6 months {m}) for patients (pts) with high-risk stage II colorectal cancer (CC). J Clin Oncol.

